# Lessons Learned from the CORE Group Polio Project and Their Relevance for Other Global Health Priorities

**DOI:** 10.4269/ajtmh.19-0036

**Published:** 2019-10

**Authors:** Henry B. Perry, Roma Solomon, Filimona Bisrat, Lisa Hilmi, Katherine V. Stamidis, Robert Steinglass, William Weiss, Lee Losey, Ellyn Ogden

**Affiliations:** 1 Department of International Health, Johns Hopkins Bloomberg School of Public Health, Baltimore, Maryland;; 2 CORE Group Polio Project/India, New Delhi, India;; 3 CORE Group Polio Project/Ethiopia, Addis Ababa, Ethiopia;; 4 CORE Group, Washington, District of Columbia;; 5 CORE Group Polio Project, Washington, District of Columbia;; 6 John Snow, Inc., Arlington, Virginia;; 7 United States Agency for International Development, Washington, District of Columbia

## Abstract

Despite numerous setbacks, the Global Polio Eradication Initiative has implemented various community strategies with potential application for other global health issues. This article reviews strategies implemented by the CORE Group Polio Project (CGPP), including pursuit of the missed child, microplanning, independent campaign monitoring, using community health workers and community mobilizers to build community engagement, community-based surveillance, development of the capacity to respond to other health needs, targeting geographic areas at high risk, the secretariat model for non-governmental organization collaboration, and registration of vital events. These strategies have the potential for contributing to the reduction of child and maternal mortality in hard-to-reach, underserved populations around the world. Community-based surveillance as developed by the CGPP also has potential for improving global health security, now a global health priority.

## INTRODUCTION

Over the past two decades, the CORE Group Polio Project (CGPP) has coordinated the engagement of non-governmental organizations (NGOs) in some of the hardest-to-reach and most resistant communities in 11 countries where polio transmission has persisted.† In 2018 alone, the CGPP programs trained more than 20,000 volunteers and health workers, reached almost six million people, and supported the vaccination of more than two million children. The contribution made by the CGPP to the Global Eradication of Polio Initiative (GPEI) has gained increasing recognition and respect. As the GPEI appears to be closing in on the achievement of what has proven to be a highly elusive goal, now is an opportune moment to address not only the lessons learned and potential legacy of the overall GPEI but that of the CGPP itself.

Previous articles in this series^1–12^ have examined various aspects of the CGPP activities and their contribution to the overall GPEI. Despite numerous setbacks, the number of wild poliovirus cases has been significantly reduced. Between January 1 and May 8, 2019, there have been only 18 documented cases of polio caused by wild poliovirus (11 from Pakistan and seven from Afghanistan) and only nine cases caused by circulating vaccine-derived poliovirus (seven from Nigeria, one from the Democratic Republic of the Congo, and one from Somalia).^13^ So, as the world transitions toward global polio eradication, what kinds of activities might the CGPP or other entities carry out that can be the beginning of a long-term contribution to other global health priorities? This is the question our article explores. One definition of “legacy” appropriate for our purposes here is the following: “something transmitted by or received from an ancestor or predecessor or from the past.”^14^ Our article discusses community-focused polio eradication strategies implemented by the CGPP and explores their potential use for other global health priorities.

## BACKGROUND

The GPEI has been an unprecedented campaign that has lasted 30 years (1989 through 2019), involving millions of volunteers, social mobilizers, and health workers who have participated in the GPEI at a cost of approximately US$22 billion dollars as of the end of 2019.^15,16^ One group of technical advisors for the GPEI considers that the most important lesson learned from the GPEI is the importance of communications and community engagement to mobilize social and community support for polio vaccination.^17^ The lessons learned and the knowledge gained by the GPEI for program implementation constitute one of the important legacies of the GPEI:

As vaccination rates increased and the proportion of missed children became increasingly confined to discrete social and socioeconomic groups, communication and social mobilization strategies were refined and targeted to reach the most vulnerable families… Through this process of mobilizing communities large and small, the polio program has developed the expertise to overcome the logistic, geographic, social, political, cultural, ethnic, gender, and other barriers to working with the most-marginalized, most-deprived, and, often, most-security–compromised children and communities.^17^

According to the GPEI’s Polio Eradication and Endgame Strategic Plan,^18^ the polio eradication program, more than any other global health program in history, has accessed the “chronically unreached, marginalized and most vulnerable populations in the world.” The CGPP played a key role in developing all of the elements described in Table 1 for reaching underserved populations.

**Table 1:** The characteristics and innovations developed to build social support for vaccination highlighted by the Global Polio Eradication Initiative^18^

1. Identification and relentless pursuit of the missed child (and newborn) 2. Identification of individuals, themes, and social pillars that can unify and motivate diverse population groups for a common goal 3. The mobilization of communities through house-by-house contact on a grand scale not only for polio immunization but also for discrete health interventions such as vitamin A supplementation, measles vaccination, anti-helminthic administration, and distribution of soap, bed nets, and oral rehydration solution packets 4. The creation of detailed local neighborhood vaccination team “microplans” and maps, and identification of locally influential people to assist in addressing those who are hesitant or resistant to immunization 5. The collection and analysis of social data at the most-local level to understand and engage effectively with the local population 6. The tracking of mobile and migrant groups and communicating with these groups while they are in transit 7. Engagement with groups while they are away from home during campaign days, such as with those attending social, cultural, or religious events (such as weddings, shrines, or festivals) 8. The use of traditional, religious, community, and civil society leaders and structures for community mobilization 9. The improvement of interpersonal skills, management, and motivation of frontline health workers 10. The development of evidence-based approaches to guide social mobilization and community engagement through ongoing, rigorous monitoring and evaluation 11. The capacity to respond to community demands for additional services beyond polio immunization 12. Engagement of communities and local civil society through other structures in addition to ministries of health 13. Mobilization of the international, national, local NGOs, and communities in high-risk areas to reach every child with polio immunization

Once global certification of polio eradication from the world has been achieved, the GPEI will no longer exist, so it is essential to plan for a transition from polio eradication to other goals and sustain the activities needed to identify and respond to circulating vaccine-derived virus. This has led to plans for a polio transition to address how investments that have been made for wild poliovirus eradication might be adopted to the post-wild poliovirus eradication context, including integration with other health efforts, and possibly shifted to other crucial health goals, including reducing the burden of disease from other vaccine-preventable diseases and from readily preventable mortality.^15^ A formalized process of polio transition planning is underway under the leadership of the Polio Legacy Management Group^19^ and has been described elsewhere.^15^ Their work is still in process, but one report on their work concludes as follows.

“The infrastructure required to eradicate polio is concentrated in many of the lowest performing low-income countries, which are the most challenging places to achieve other health objectives. Now is the time to determine how this massive infrastructure for polio eradication can be sustained and repurposed, for example, measles eradication and immunization system strengthening.”^15^

Other potential beneficiaries of repurposing the existing polio eradication infrastructure include regional malaria control and elimination as well as health systems strengthening initiatives of the Global Fund to Fight AIDS, Tuberculosis and Malaria; GAVI, the Vaccine Alliance; and the United States Agency for International Development (USAID) Global Health Security Agenda.

Many of the lessons learned from the CGPP arose from the CGPP’s work with very specific population groups, namely, those who have been chronically unreached, marginalized, and highly vulnerable. Experience has shown that these populations are likely to push back against top–down vertical programs that focus on narrow disease-specific priorities unless efforts are made to recognize these people as a valued resource whose own needs and priorities for improved health are also addressed.

Specific lessons learned include the following.

*The social mobilization network (SMNet) is a powerful tool for achieving public health priorities*. The United Nations Children's Fund (UNICEF), the CGPP, and Rotary International developed the SMNet in 2000 in Uttar Pradesh and Bihar, India. SMNet consists of five key strategies.^20^

1. Leveraging effective partnerships with international agencies, government agencies, civil society (including professional associations and universities), NGOs, and local community organizations down to the village level, often requiring the provision of other priority basic health services to gain local acceptance and ownership
2. Human resource capacity building, including the recruitment and training of local workers (called community mobilization coordinators)
3. Evidence-based planning at the local level (referred to as “microplanning”) based on comprehensive mapping of all households, development of field books with lists of all children younger than 5 years of age, their age, and vaccination status
4. Communication and social mobilization for behavior change using Community Mobilization Coordinators, house-to-house interpersonal communication, group counseling sessions, engagement with “influencers” such as religious leaders, as well as the use of print and mass media to convey key messages
5. Monitoring, evaluation, and supportive supervision, including household surveys, concurrent monitoring of immunization activities (allowing for in-course corrective actions during campaigns), and review of data from the health information system.

SMNET was implemented in the hardest-to-reach areas and underserved communities where there was deep-rooted resistance to immunization. SMNET contributed to a decline in refusals in both Bihar and Uttar Pradesh to less than 1% of households.^20^ Furthermore, full routine immunization coverage in areas defined by the GPEI as being at high risk for polio transmission in Uttar Pradesh increased from 36% in 2009 to 81% in 2016 and in Bihar from 54% in 2009 to 89% in 2016.

*Cross-border initiatives are needed for controlling cross-border transmission of infectious diseases*. In 2003, the CGPP initiated the Cross-Border Initiative in the Horn of Africa, first in Ethiopia and then later in South Sudan, Kenya, Somalia, Uganda, and the Democratic Republic of the Congo. The primary components of the Cross-Border Initiative are the establishment of periodic cross-border meetings and cross-border health committees to coordinate vaccination efforts around the many porous border crossing points in the Horn of Africa. These meetings and committees established relationships between health workers on both sides of international borders, helped to synchronize national and sub-national immunization days, created border vaccination stations (called Transit Vaccination Posts), and mapped border health facilities that can help people crossing regional borders to be protected from and to not carry diseases across the border that can cause outbreaks. In addition, CGPP developed links with individuals in communities, such as barbers,‡ to alert health workers to newly arrived mobile and nomadic populations, thereby improving microplans and acceptance of immunization.

*Community-based surveillance can be a useful complement to facility-based surveillance in hard-to-reach populations*. Community-based surveillance programs identify a network of local volunteer informants who are likely to hear about a case of acute flaccid paralysis in their community. In addition, the CGPP implemented surveillance for two other vaccine-preventable diseases—measles and neonatal tetanus.

Unlike a standard surveillance system, which relies on trained paid health professionals working in health facilities to report diseases from patients who present at health facilities for treatment, a community-based surveillance system trains local community members such as traditional healers, traditional birth attendants, elders, community leaders, religious leaders, teachers, social mobilizers, community health workers, and others to recognize cases of targeted diseases and how to report them. This ancillary approach complements existing facility-based surveillance systems by making it possible to identify cases earlier and also to identify cases among those who might choose alternative forms of health care rather than go to a formal health facility.

This system provided a useful alternative in South Sudan during the recent conflict when many of the formal health facilities were destroyed, closed, or became dysfunctional as described in another article in this series.^11^ In 2018, the CGPP adopted community-based surveillance to support disease surveillance for the Global Health Security Agenda, targeting a larger number of primarily zoonotic diseases in Kenya and Ethiopia (anthrax, brucellosis, echinococcosis, leptospirosis, rabies, trypanosomiasis, and Rift Valley fever). The CGPP project areas are also able to assist with preparedness and response to public health threats such as a polio outbreak.

*Independent campaign monitoring (ICM) is essential for priority public health programs*. The CGPP collaborated with the World Health Organization WHO and the ministries of health in Angola in 2000 and later in South Sudan in 2012 to introduced ICM. Before the introduction of ICM, campaign coverage and quality were measured using “administrative data” (data obtained from reports of health workers regarding the number of doses given) and then calculating the percentage of children vaccinated in the population using the best available estimate of the population of children in the target population. Administrative coverage data was often unreliable because of 1) a lack of reliable census data from which to obtain an accurate denominator and 2) questionable accuracy of tally sheets of the number of children vaccinated. Administrative data often promoted a false sense of security and accomplishment as districts would sometimes report rates of more than 100% when in reality, the coverage was much lower, as evidenced by a massive wild poliovirus outbreak in Angola in 1999 with more than 1,000 cases reported. The first independent post-campaign coverage survey conducted in Angola in 2000 found a campaign coverage of 71% and gave the program a much more accurate sense of the campaign quality and of the amount of improvement required to achieve interruption of transmission of wild poliovirus. The accuracy of the data provided by ICM contributed to the initial interruption of wild poliovirus transmission in 2001.

*The CGPP secretariat model is an effective way of mobilizing NGOs and civil society*. Although NGO health projects often have significant community-level impact in limited geographical areas, their efforts are often isolated and balkanized (i.e., they can be uncoordinated, small entities that are even sometimes working at odds with each other) and, therefore, not conducive to the achievement of large-scale global initiative such as polio eradication. The CGPP used a secretariat model at the country level to establish NGO partners (these are listed in Appendix II; table 2 of the first article in this series^1^).

The organization that has evolved to manage and support the CGPP activities—namely, the CGPP Global and Country Secretariats working with international and national NGOs who in turn work with civil society—has been a powerful and effective mechanism for engaging communities in polio eradication activities. It has also been a highly efficient and effective way for international donors to channel financial support for programming that will reach vulnerable and hard-to-reach populations. The secretariat model was used by USAID in 2007 to support the global efforts of WHO, the International Federation of the Red Cross, and the CORE Group to prepare for pandemic influenza, which at that time had become a global health emergency.

*NGOs are a useful resource for complementing what governments can provide to address health priorities*. The CGPP was implemented entirely through NGOs—international NGOs working with local NGOs, but coordinated with government and UN implementing partners (WHO and UNICEF) and integrated with the local ministry of health immunization program. The achievements of the CGPP would not have been possible by working only with government structures because of a lack of adequate government staffing at the periphery and a lack of interest and capacity among government workers at the periphery to manage this kind of work.

The NGO capacities that have been developed in the process of carrying out CGPP activities, the human resources that have been developed (technical expertise and capacities of community workers and their managers) could be applied to other health priorities rather than letting them dissipate and wither from lack of continued support. Maintaining the NGO network that has been established and, perhaps, even expanding it more broadly could be a key resource for health improvement generally but also specifically for surveillance and global health security. This network was used in 2007 to prepare for a global pandemic influenza epidemic that appeared to be emerging at that time, and of course that threat still remains.

*Relentless pursuit of the missed child (or of children in need of essential services) is necessary for child health programs to achieve optimal results*. This requires identifying who these children are and mobilizing communities to help ensure that essential services reach these children.

*Local registration of vital events and child registries are feasible and useful*. Using community mobilizers to register births and using these registers to identify children in need of immunizations during their first year of life and their household location made it possible to track children and expand the coverage of polio and routine immunizations in low-performing areas, including the coverage of the birth dose for polio immunization. It was also possible to identify pregnant women and encourage them to go for antenatal care. This required community mobilizers regularly visiting all homes. Registration of deaths can be readily incorporated into the system.

*Promotion of services in the home is necessary in part to identify children who are in need of essential services, provide referral, look for outbreaks of infectious diseases, and register vital events (births and deaths)*. In addition to ensuring every home was visited during semi-annual supplemental immunization campaigns, home visiting provided the opportunity to provide oral polio vaccine for those who failed to come to a service delivery point for vaccination—essential for reaching every last child. Reaching every home on a regular periodic basis (at least every 2 months) also provided the opportunity for health promotion messaging—especially around breastfeeding and water, sanitation, and hygiene—and surveillance of acute flaccid paralysis.

*Provision of a broader set of basic and essential services that respond to community-felt needs helps secure buy-in from local communities for disease-control activities (such as polio eradication) that are not community priorities*. Expanding the range of services provided beyond polio and routine immunization services (such as antenatal care, nutrition screening, blood pressure screening, and provision of health camps to treat malaria and other acute conditions) was essential for reaching populations who had become resentful of the ongoing solitary focus on polio.

**What are global health priorities that the CGPP experience could contribute to?** There are now 15 million people around the world dying from readily preventable or treatable conditions: pregnancy-related deaths, infants in the process of being delivered who are born dead (stillbirths) and children who die before reaching the age of 5 years,^21^ and others (including adults) dying of HIV-related illnesses, malaria, or tuberculosis.^22–24^ Aside from this, hypertension kills an estimated 9.4 million people annually worldwide, about as many as all infectious diseases combined.^25^ Hypertension and other important non-communicable diseases such as obesity, diabetes, and mental illness along with neglected tropical diseases have great potential for control through community engagement and community-based service delivery with community-level workers.^26^ At least half of the world’s population still does not have full coverage of essential health services and paying for health care is a major financial burden for 12% of the world’s population.^27^ All UN Member States have agreed to try to achieve Universal Health Coverage by 2030, as part of the Sustainable Development Goals.^27^ Community-based approaches such as those mastered by the CGPP can make important contributions to these global health priorities.

The global goal of ending preventable child and maternal deaths by achieving rates of child and maternal mortality that were achieved in the industrialized countries in 1950,^28^ in particular, can benefit from a broader application of the approaches elaborated in the articles in this series. Its achievement by the year 2030, as envisioned, will require a doubling of the rate of decline of under-five and maternal mortality.^29^

Applying the CGPP approach of using community-level workers to visit all homes on a regular basis would make it possible to address the leading local causes of maternal, perinatal, neonatal, and child deaths through the implementation of evidence-based interventions for improving reproductive, maternal, neonatal, and child health, the importance of which has been highlighted by others.^30,31^ Further potential benefits of routine systematic home visitation include the feasibility of using these visits to register vital events and conduct surveillance for vaccine-preventable diseases and other disease outbreaks. Thus, building on this important element of the CGPP approach in resource-constrained, high-mortality settings has the potential to serve as a lasting legacy of the CGPP approach. In addition, the CGPP approach of community engagement, which of course also builds on the experience of many other organizations—especially those in the NGO sector—could be applied to underserved and high-risk urban/periurban populations. The results of a pilot experience using some of the elements of the CGPP approach by an NGO in the slums of Freetown, Sierra Leone, have recently been reported.^32^

The Ebola outbreak response was built on the backbone of polio eradication efforts in Nigeria, averting what could have been a much bigger catastrophe than occurred in Guinea, Liberia, and Sierra Leone at the time of the 2014–2016 Ebola epidemic.^33^ Polio program infrastructure has strengthened disease outbreak response to Marburg hemorrhagic fever, dengue fever, measles, anthrax, and shigella across the WHO Africa region,^34^ and broader application of frequent contact with all homes as used in the CGPP could have made outbreak detection earlier.^35^ Thus, the GPEI and, in particular, the methods used by the CGPP have important applications to the development of global health security and the early control of outbreaks such as Ebola and influenza-related infections and zoonotic diseases (as already mentioned), among others or the response to humanitarian emergencies and disasters.^36^

**The potential of community engagement, strengthening community platforms, and promotion of community ownership**. The great majority of current evidence-based interventions to prevent deaths among mothers and their young offspring—such as self-administration of misoprostol during the third stage of labor by women who deliver at home, home-based neonatal care, and integrated community case management of serious childhood illness—can be implemented in the community by community-level workers.^31,37^ However, the population coverage of these high-impact interventions is surprisingly low except for immunizations and vitamin A supplementation. In the 74 countries where 97% of the deaths of children and mothers occur, the median national population coverage is less than 50% for one-third of these interventions. Many of these interventions need to be delivered around the time of birth or at the time of an acute illness.^38^

The public health community is only beginning to learn how to work effectively with communities to achieve the full potential of health systems for improving health. Building a strong long-term sustainable community platform that promotes community ownership, engages communities, and uses community-level workers has the potential to contribute to ending preventable child and maternal deaths and for achieving Universal Health Coverage.^30,31,39^ The approach of the CGPP, if applied more broadly, could be an important resource for meeting these global health goals in the most difficult-to-reach and vulnerable populations around the world. If the experience of the CGPP contributes to a broader recognition of the importance of building a stronger community platform for health systems, then this could well become the most important legacy of the CGPP.

## CONCLUSION

The Global Polio Eradication Initiative, more than any other global health program in history, has accessed the “chronically unreached, marginalized and most vulnerable populations in the world.”^18^ This is a strength that should be used to address other health priorities.

The CGPP has been working in areas where polio was difficult to eliminate—where people were hard to reach, health services were weak, and where immunization coverage was low. These are also areas with high maternal and child mortality. Therefore, transitioning the target from polio eradication to ending preventable child and maternal deaths and continuing to use the NGO networks for community-based surveillance for identifying and responding to disease outbreaks and for responding to emergencies would be a readily achievable shift because the strengths of the CGPP could be applied and the results measured.

NGO networks have the potential to contribute to decreasing child and maternal mortality rates through the application of the following actions that have contributed to significant decreases in the transmission of wild poliovirus worldwide:

1. Support for community-level workers who can promote healthy household behaviors as well as health facility utilization when warning signs develop
2. Support for the existing CGPP community-based systems and their coordination with health facilities to promote outreach vaccination and to identify cases of vaccine-preventable diseases
3. Continuation of close coordination between the CGPP community-based systems and the formal health services system because injectable immunizations provided by facility-based workers who are part of outreach teams will be necessary, including for the transition from oral polio immunization to injectable inactivate polio vaccine until polio has been eradicated
4. Support for and strengthening of existing CHW programs with lessons learned from the CGPP, including CHW-led behavior changes using interpersonal communication, consistent and repeated contact, and modeling of positive behaviors
5. Application of these successful community-based methods of engagement in underserved and high-risk urban/periurban populations
6. Continuation and expansion of the secretariat model to coordinate NGO collaboration

Through the aforementioned activities, it would be feasible to expand the approach with the activities listed in the following paragraphs to achieve broader public health benefits beyond polio:

1. Fostering the capacity of communities to take ownership of their health problems by helping them understand what the levels of mortality of mothers and children are in their community, the degree to which mortality has been declining (or not), and how they can contribute to further reductions in mortality
2. Monitoring of vital events (deaths as well as births) through systems of community collaboration
3. Introduction of verbal autopsies through community collaborations to help communities understand who is dying of what and actions that could be undertaken by the community to prevent similar deaths in the future; building strong community platforms to achieve Universal Health Coverage where facilities are not readily accessible and to increase the effectiveness of health systems in improving population health—particularly by reducing the number of deaths from readily preventable or treatable conditions—will require adopting and expanding the strategies developed by the CGPP for polio eradication into the mainstream of activities for routine services, including routine immunizations.^40^

## Footnotes

† A complete listing of these NGOs is located in Appendix II of the first article in this series.^1^

‡ The CGPP encourages barbers in India to communicate with fathers on the importance of vaccination and in Nigeria when newborns are brought for the Islamic rite of hair cutting on the seventh day of life.
